# State Transitions During Discrimination Learning in the Gerbil Auditory Cortex Analyzed by Network Causality Metrics

**DOI:** 10.3389/fnsys.2021.641684

**Published:** 2021-04-22

**Authors:** Robert Kozma, Sanqing Hu, Yury Sokolov, Tim Wanger, Andreas L. Schulz, Marie L. Woldeit, Ana I. Gonçalves, Miklós Ruszinkó, Frank W. Ohl

**Affiliations:** ^1^Center for Large-Scale Intelligent Optimization and Networks, Department of Mathematics, University of Memphis, Memphis, TN, United States; ^2^College of Computer Science, Hangzhou Dianzi University, Hangzhou, China; ^3^Department of Medicine, University of California, San Diego, La Jolla, CA, United States; ^4^Leibniz Institute for Neurobiology (LIN), Magdeburg, Germany; ^5^Alfréd Rényi Institute of Mathematics, Budapest, Hungary; ^6^Faculty of Information Technology and Bionics, Pázmány Péter Catholic University, Budapest, Hungary; ^7^Institute of Biology, Otto von Guericke University, Magdeburg, Germany; ^8^Center of Behavioral Brain Science (CBBS), Magdeburg, Germany

**Keywords:** auditory cortex, electrocorticogram, discrimination learning, granger causality, new causality, graph theory, percolation, state transition

## Abstract

This work studies the evolution of cortical networks during the transition from escape strategy to avoidance strategy in auditory discrimination learning in Mongolian gerbils trained by the well-established two-way active avoidance learning paradigm. The animals were implanted with electrode arrays centered on the surface of the primary auditory cortex and electrocorticogram (ECoG) recordings were made during performance of an auditory Go/NoGo discrimination task. Our experiments confirm previous results on a sudden behavioral change from the initial naïve state to an avoidance strategy as learning progresses. We employed two causality metrics using Granger Causality (GC) and New Causality (NC) to quantify changes in the causality flow between ECoG channels as the animals switched to avoidance strategy. We found that the number of channel pairs with inverse causal interaction significantly increased after the animal acquired successful discrimination, which indicates structural changes in the cortical networks as a result of learning. A suitable graph-theoretical model is developed to interpret the findings in terms of cortical networks evolving during cognitive state transitions. Structural changes lead to changes in the dynamics of neural populations, which are described as phase transitions in the network graph model with small-world connections. Overall, our findings underscore the importance of functional reorganization in sensory cortical areas as a possible neural contributor to behavioral changes.

## 1. Introduction

There is a growing body of literature on the neural basis of learning and memory formation over various sensory modalities in humans and animals (Goldstone, 1998; Seger and Miller, 2010; Chapuis and Wilson, 2012; Aizenberg and Geffen, 2013). For example, in auditory learning there is ample experimental evidence of learning-induced plasticity in various neuronal structures including primary and higher-order auditory cortex (Eggermont et al., 1983; Weinberger et al., 1984; Ohl and Scheich, 1996; Villa et al., 1998, 1999a; Fritz et al., 2003; Weinberger, 2004, 2007; Plakke et al., 2013; Grosso et al., 2015, 2017; Cambiaghi et al., 2016; Concina et al., 2018), corpus striatum (Znamenskiy and Zador, 2013), and prefrontal cortex (Romanski et al., 1999; Funamizu et al., 2013; Concina et al., 2018). In such neural structures, the responses of single neurons and population of neurons to relevant spectral, temporal, or spectrotemporal features of stimuli undergo changes when these features attain behavioral or cognitive relevance during learning or specific task scenarios (Villa et al., 1999b; Machens et al., 2004; Ohl and Scheich, 2005; Bar-Yosef and Nelken, 2007; Schreiner and Winer, 2007; Rabinowitz et al., 2013; Meyer et al., 2014; Weinberger, 2015).

However, learning is not a singular continuous process that takes an individual from a naïve state to a learned state, rather it constitutes a set of discernible subprocesses that typically take the subject through several distinguishable phases, even for the simplest learning scenarios (Cambiaghi et al., 2016; Deliano et al., 2016). As these phases evolve, stimulus processing in the sensory nervous system may change in association with changes in the cognitive state.

Here we investigate the behavioral state changes and corresponding neuronal changes in the auditory cortex during a standard auditory-cued Go/NoGo task in a shuttle box. The shuttle box Go/NoGo discrimination task is a well-established experimental paradigm to investigate auditory learning (Ohl et al., 1999; Wetzel et al., 2008; Deliano et al., 2009; Happel et al., 2014; Schulz et al., 2016) and the formation of cognitive categories in rodents (Ohl and Scheich, 1997; Wetzel et al., 1998; Ohl et al., 2001, 2003). In this paradigm, the animal is placed in a two-compartment cage (shuttle box) with a little hurdle separating the two compartments. The animal is trained to cross this hurdle in response to a specific auditory signal and to stay in the current compartment in response to another signal, in order to avoid a mild aversive electrical stimulation (“foot shock”) via the floor grid. This is achieved by consequently preceding the foot shock with the acoustic stimulus (to provide the possibility for prediction), and by terminating the shock upon the desired behavioral response, i.e., crossing the hurdle. Previous work has shown that this type of learning is determined by complex behavioral processes, involving at least three different stages that occur in sequence:

1. *Conditioning:* In the first stage, the animal learns that the sensory stimulus predicts the foot shock. The behavioral process and its underlying mechanisms are akin to Pavlovian conditioning in which a conditioned stimulus (CS) is learned to have predictive power for the occurrence of an unconditioned stimulus (US).
2. *Escape Strategy:* In the second stage, the animal learns that the aversive effect of the foot shock can in fact be terminated by crossing the hurdle (escape strategy). Here the animal learns that an appropriate behavior can have desirable effects on the reinforcement scenario (e.g., a reduced duration of an aversive event), which is an important element of the classical concept of instrumental or operant conditioning.
3. *Avoidance Strategy:* In the final stage, the animal learns that the aversive foot shock can be avoided altogether, if it crosses the hurdle fast enough after onset of the sensory signal. In the avoidance phase, the animal acquires a new behavioral strategy by re-evaluating its hitherto existing behavioral strategy (escape) and replacing it by a new one (avoidance).

The transition from the escape to the avoidance strategy represents a behavioral and cognitive state change (Ohl, 2015; Kozma and Freeman, 2016). The underlying neuronal mechanisms of such state change are not well-understood. In particular, it remains to be seen how the local interactions between neural assemblies change after the switch to avoidance strategy.

Functional connectivity in various networks has been widely analyzed using causality measures. *Granger causality (GC)* has been successfully applied to identify the directional influence of system components in many different fields, such as economics (Chen et al., 2011), climate studies (Kodra et al., 2011), genetics (Zhu et al., 2010), and neuroscience (Ding et al., 2006; Wang et al., 2008; Bressler and Seth, 2011; Gao et al., 2011; Ge et al., 2012; Barnett and Seth, 2014; Seth et al., 2015; Cambiaghi et al., 2016). Granger formulated his approach to causal analysis (Granger, 1969), following Wiener's insight (Wiener, 1956). The key idea can be summarized as follows: Time series *X*_1_ has GC relationship with time series *X*_2_, if the prediction accuracy of *X*_2_ can be improved by using past values of *X*_1_. Granger causality has many successful applications, but it has limitations in recovering causal relationships in complex networks (see Spirtes et al., 2000; Pearl, 2009). This is especially true in highly convoluted brain networks with circular causality interactions, in the presence of common-mode observation noise, field effects, and volume conduction (see Friston et al., 2014; Pesaran et al., 2018; Pascual-Marqui et al., 2021). To address some of the limitations of Granger causality, Hu et al. (2011) proposed the *New Causality (NC)* method, which considers the proportion that Y occupies among all contributions to predict X. As demonstrated by a number of applications, NC is often more robust than GC in revealing causal relationships in data (Hu et al., 2011, 2012). NC and GC methods have been successfully applied in motor imagery to identify causal flows among regions Cz, C3, and C4, whereas NC shows significantly improved classification rate, as compared to GC (see, e.g., Hu et al., 2015; Kozma and Hu, 2015).

In this paper, we describe electrocorticographic (ECoG) data recordings using a 5 × 4 electrode array centered on the top of the primary auditory cortices of seven Mongolian gerbils, while they perform an auditory Go/NoGo discrimination task using two distinct frequency modulated (FM) tones as conditioned stimuli, CS+ and CS−. Based on previous results, in particular (Ohl et al., 1999, 2000a,b, 2001, 2003; Freeman, 2000), we focus our analysis on the beta/gamma band (20–80 Hz). We extend on preliminary results reported in Kozma et al. (2016a,b).

Transitions in the learning performance can be described using various methods. This work uses the approach called *d*′ (d-prime), which is based on signal detection theory (Deliano et al., 2009; Happel et al., 2014). As an alternative criterion, conditioned response (CR) behavioral signature be used as well, in which case transition is detected when the CR rate exceeds a given threshold the first time. We use *d*′-based discrimination in the present work, following decades-long tradition of behavioral and physiological analysis. We identified channel pairs with inverse bidirectional causality flow for the two tone patterns representing CS+ and CS−. The results show that learning produces statistically significant increase in the number of causal pairs, as the gerbils transit from the escape strategy to the avoidance strategy. This, in turn, is an indication of adaptive reorganization in cortical networks. Our results indicate the emergence of spatial patterns of causality flow as learning progressed. The increased causality flows are detectable both by GC and NC measures, while NC appearing to be more robust than GC in terms of extracting spatial patterns across experiments with various animals.

We introduce a mathematical model of neurodynamical processes across the cortical layers, based on *percolation processes* in graphs and networks (Balister et al., 2006; Kozma et al., 2016b; Janson et al., 2019). Of special interest is the adaptation of brain networks during the learning process. Brain networks have been intensively studied in recent decades, identifying structural, functional, and effective connectivity graphs (Bollobás et al., 2010; Bressler and Seth, 2011; Bullmore and Sporns, 2012; Haimovici et al., 2013; Kozma and Freeman, 2016).

This study develops a neurodynamic theory based on the hierarchy of *Freeman K sets*, corresponding to increasing complexity of structure, dynamics, and function (Freeman et al., 1975; Freeman, 1991). The analysis uses basic building blocks of neurodynamics, including excitatory and inhibitory neural populations, called Freeman KI sets, which can generate non-zero fixed point dynamics for a sufficient level of mutual interaction. We also study interacting excitatory and inhibitory populations (Freeman KII sets), which may exhibit limit cycle oscillations in response to previously conditioned sensory stimuli (Kozma and Freeman, 2003, 2009). The implementation of KI and KII networks dynamics using percolation processes in 2-dimensional models of the cortical neuropil demonstrate potential benefits of the proposed network theory approach in the interpretation of the experimental findings, especially when describing the emergence of common-mode oscillations in neural cell assemblies as the result of the learning process. We extend earlier results on stability analysis by Sokolov and Kozma (2014) and on cortical phase transitions between fixed-point and limit cycle oscillations by Kozma et al. (2016a,b) and Kozma and Freeman (2016). The introduced results underscore the importance of functional reorganization in early sensory areas of rodents in discrimination learning and identify inverse causality flows as possible neural signatures of behavioral changes.

## 2. Materials and Methods

### 2.1. Experimental Paradigm

The behaviorial paradigm is summarized here; for details, see Ohl et al. (2001). A rectangular electrode array with 20 electrodes was implanted above the auditory cortex on top of the dura mater; for the spatial arrangement of surface array electrodes (see Supplementary Figure 2). In addition, a wire bundle of 8 depth electrodes was implanted into the ventral striatum. In the present study only the signals from the auditory cortex were analyzed. Gerbils were exposed to two different types of tones: linear rising frequency modulated tones (FM 2–4 kHz, duration 200 ms) and falling FM (FM 2–4 kHz, duration 200 ms). FM tones were presented not as single tones but in short sequences, with 300 ms pauses, yielding an inter-stimulus-interval of 500 ms. This allowed to produce a sufficiently long auditory stimulus while simultaneously keeping the frequency-modulation slope of each segment sufficiently high for robust learning (Ohl et al., 1999, 2000b). Every sequence consisted solely of rising or falling FM signals, but not of a mixture of alternating rising and falling FM signals.

The gerbils were trained in a shuttle box to move from one side of the shuttle box to the other, by crossing a small hurdle, when the rising FM tone (CS+) occurred (Go trial). The falling FM tone (CS−) served as the NoGo signal. If the animal crossed the hurdle within a time period of 6 s after CS+ onset (*hit*) the tone was switched off, the trial terminated and after an inter-trial interval of 25–30 s, the next trial started. If the animal missed to cross the hurdle within the 6 s window (*miss*) in a Go trial, it received a mild electrical foot shock via the metallic grid floor of the shuttle box. This resulted in a forced escape response to the other side of the box, after which both shock and tone were switched off (end of trial).

For the falling FM (CS−) animals were supposed to stay within the presently occupied compartment of the box for at least 10 s, which corresponded to a *correct rejection* and terminated the trial. If animals did change compartments during such a NoGo trial (*false alarm*), they also received a mild electrical foot shock directly after hurdle crossing and the trial ended. One training session, which corresponded to one training day, consisted of 96 trials (48 Go and NoGo 48 trials). The pseudo-randomized trial sequence was identical for every day and was coded in an offline list (based on a Gellermann sequence). Trials, in which the signal was contaminated with artifacts, were removed from further analysis. Consequently, the effective number of trials of a session could be different from 96.

We calculated for every session a d-prime *d*′ value (signal detection theory) based on the correct CR (*hit*) and incorrect CR (*false alarm*). A d-prime value exceeding 1 indicates statistically significant change (Deliano et al., 2009; Happel et al., 2014). We used d-prime to identify two stages of the experiments, Stage 1 and Stage 2, always starting with Stage 1. If on three consecutive sessions the condition *d*′ > 1 was satisfied, we marked the first of these sessions as the first session of Stage 2. Therefore, the identification of Stage 1 and Stage 2 was based on the behavior of the animals.

### 2.2. Metrics for Causality Analysis of Gerbil Data

We analyzed causal flows between electrodes implanted over the auditory cortex of seven gerbils; for details, see Supplementary Material. Granger Causality (GC) is based on the notion that if historical information of time series *X*_1_ significantly improves the prediction accuracy of the future of time series *X*_2_, then time series *X*_1_ has causal relationship with *X*_2_ in the sense of GC (see Granger, 1969). Granger Causality has been widely applied to identify the directional influence of system components in many different fields, including neuroscience (e.g., Ding et al., 2006; Wang et al., 2008; Bressler and Seth, 2011; Gao et al., 2011; Ge et al., 2012). In addition to GC, we use New Causality (NC) proposed by Hu et al. (2011, 2012). New Causality generalizes the concept of GC, such that it considers the proportion that *X*_1_ occupies among all contributions to predict *X*_2_. As demonstrated by a number of applications, NC may provide additional insight beyond GC in revealing details of causal relationships in data, as shown by Hu et al. (2015) and Kozma and Hu (2015). We use the following notations for causal flow from *X*_1_ to *X*_2_ (*X*_1_ → *X*_2_):

$$\begin{array}{l} n_{X_{1}\to X_{2}}^{CS+}\mathrm{and}n_{X_{1}\to X_{2}}^{CS-}denote causal flow using NC\\ \text{ for CS+ and CS− trials},\text{respectively};\\ F_{X_{1}\to X_{2}}^{CS+}\mathrm{and}F_{X_{1}\to X_{2}}^{CS-}denote causal flow using GC\\ \text{ for CS+ and CS− trials},\text{respectively}. \end{array}$$

If the experimental conditions involve two stimuli, e.g., CS+ and CS−, the notion of *inverse causality* may be of benefit. When determining inversely causal pairs, we seek pairs (*X*_1_, *X*_2_) with the property that the causal flows between *X*_1_ and *X*_2_ are of the opposite direction for CS+ and CS− trials, respectively. Specifically, for NC method, we collect all pairs satisfying condition:

(1) $$\begin{array}{r} \left(n_{X_{1}\to X_{2}}^{CS+}-n_{X_{2}\to X_{1}}^{CS+}\right)\times \left(n_{X_{1}\to X_{2}}^{CS-}-n_{X_{2}\to X_{1}}^{CS-}\right)<0. \end{array}$$

Similarly, for GC method, we collect all pairs satisfying condition:

(2) $$\begin{array}{r} \left(F_{X_{1}\to X_{2}}^{CS+}-F_{X_{2}\to X_{1}}^{CS+}\right)\times \left(F_{X_{1}\to X_{2}}^{CS-}-F_{X_{2}\to X_{1}}^{CS-}\right)<0. \end{array}$$

Using the above conditions, we determine inverse causality pairs for each animal in each session, where the causal flow values are averaged over all CS+ or CS− trials in the specific session. Using the GC and NC measures, we develop the following algorithm to identify possible learning-induced changes in the cross-channel causality flow:

1. Collect those pairs that satisfy condition (1) for NC or (2) for GC, i.e., channel pairs that showed significant inverse causality flow in CS+ vs. CS− trials; these are called *collected pairs*.
2. Identify a subset of *collected pairs*, which appeared only after the animal switched to the avoidance strategy during the learning process. These pairs are referred to as *core pairs*.
3. Analyze the causality flows for *collected pairs* in all sessions, we introduced a statistical index (classification measure) to characterize the discrimination performance of the given pair of electrodes between CS+ and CS− trials.
4. Analyze the evolution of the spatial distribution of *collected pairs* and *core pairs* in the 5 × 4 electrode array.
5. Determine specific cortical activity patterns, which may serve as mesoscopic neural correlates of the learning process.

In section 3, we determine causality indices (NC and GC) for all electrode pairs band-passed evoked potentials filtered over the gamma band (20–80 Hz). First we illustrate the algorithm for one representative animal (Gerbil 1), then we present a comprehensive group analysis for seven animals.

## 3. Results

### 3.1. Causality Analysis During Auditory Discrimination Learning in Gerbil 1

#### 3.1.1. Collected Pairs With Inverse Causality Relationship

**Figure 1:**
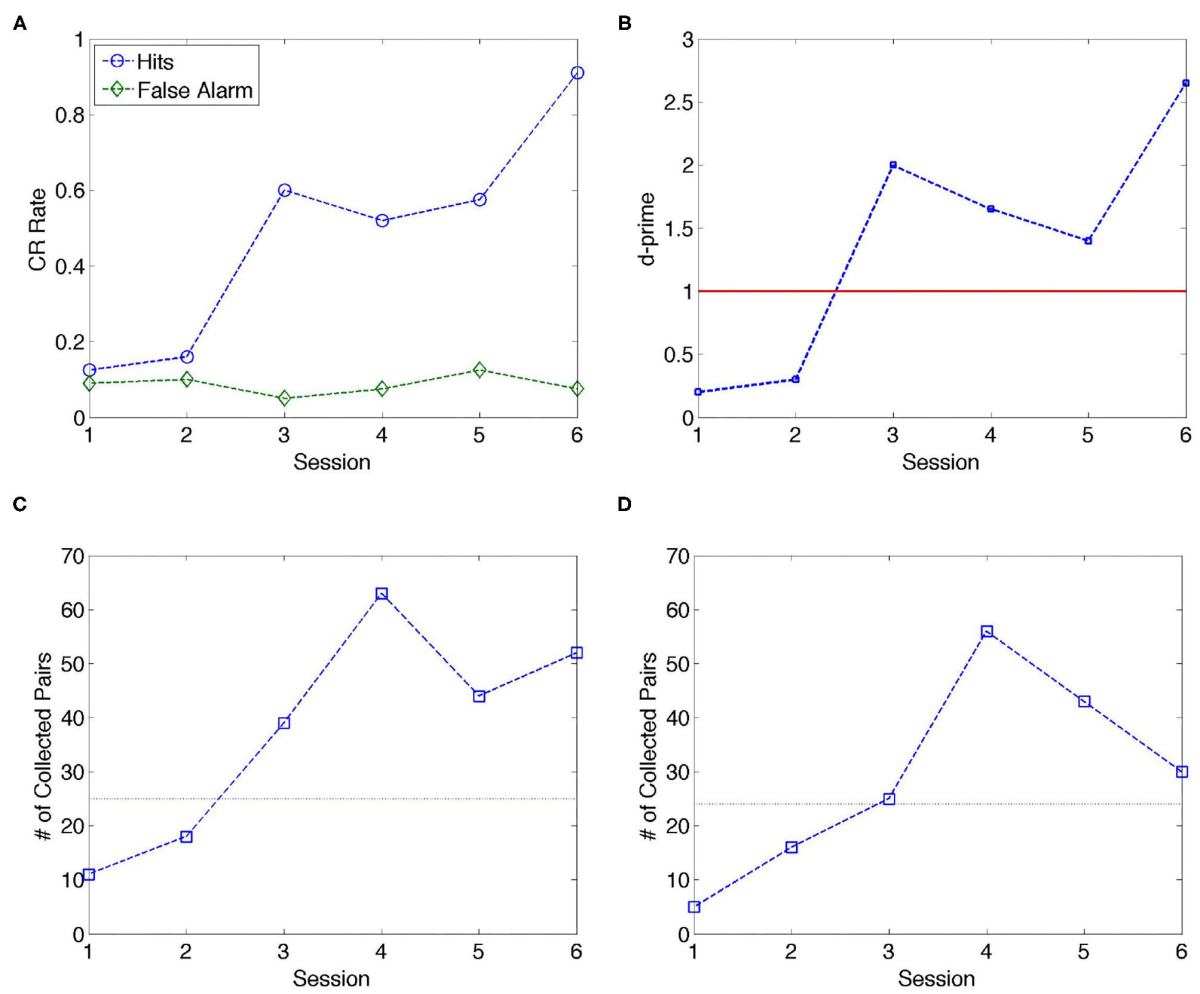
Analysis of data over six training sessions for one animal (Gerbil 1). **(A)** Conditioned response (CR) rate per session. From Session 3 onwards, the discrimination performance of the gerbil markedly improves in terms of *hits*; the CR rate corresponding to *false alarms* maintains a low value around 0.1 through the training sessions. **(B)** *d' (d-prime)* quantifies detection sensitivity independently of the response bias of the animal; *d*′ > 1 indicates better than chance level; from Session 3, the gerbil performed significantly above chance level. **(C,D)** Number of collected pairs using the inverse causality criterion determined by NC and GC methods, respectively. The dashed line is drawn to guide the eye in separating Stage 1 (Sessions 1 and 2) and Stage 2 (Sessions 3–6).

Data for Gerbil 1 include six daily training sessions. Figure 1A shows the behavioral response of Gerbil 1 with *hits* (blue) and *false alarms* (green) evolving during the training sessions; the conditioned response (CR) rate indicates the percentage of *hits* and *false alarms* per session. There is a marked increase in the CR rate of the *hits* starting from day 3. These results are in accordance with previous research conducted on gerbils using auditory learning (Ohl et al., 2001). Causality flow analysis has been conducted using 18 channels with this animal, while 2 channels (#10 and #26) have been excluded from this study due to artifacts (see Supplementary Table 1), last column of the first row. As a result, a total of 153 channel pairs have been analyzed for inverse causality flow.

According to Figure 1C, the number of collected pairs per session was consistently higher in sessions (S3–S6) after the animal switched to avoidance strategy, as compared to naïve sessions before the switch (S1 and S2) for NC analysis. Similar conclusion can be drawn from Figure 1D in the case of GC criteria. In Table 1, sessions corresponding to the escape behavior (S1 and S2) are marked as Stage 1, while sessions after the switch to avoidance stage are marked as Stage 2. Table 1 displays the mean values of the collected pairs in Stages 1 and 2, respectively, clearly indicating the increased number of collected pairs in Stage 2. The observation that the number of the collected pairs is larger in Stage 2 than in Stage 1 gives rise to the hypothesis that the number of causal pairs obtained using NC and GC methods contain relevant information on the cognitive state of the animal. Next, the consistency of this hypothesis is evaluated.

**Table 1:** Number of collected pairs in Gerbil 1 Sessions S1–S6; NC and GC methods.

	**Stage 1**		**Stage 2**			
	**S1**	**S2**	**S3**	**S4**	**S5**	**S6**
NC	11	18	39	63	44	52
GC	5	16	25	56	43	30
Mean of stage (NC)	14.5		49.5			
Mean of stage (GC)	10.5		38.5			

#### 3.1.2. Core Pairs Marking the Transition to Avoidance Phase

From the pool of all collected channel pairs, we sought to identify those pairs that emerged as potential predictors of CS contingency in Stage 2; i.e., the pairs that can be used to discriminate CS+ vs. CS- trials after the transition to the avoidance phase. To this aim, we defined *core pairs* that were collected in every session of Stage 2, but were absent during the sessions of Stage 1. Table 2 shows the example of (C21, C23) as a core pair for the NC method, while (C1, C19) is a core pair using GC approach. Indeed, according to Table 2, NC values are always larger from C21 to C23 than from C23 to C21 in the case of CS+ trials, while NC values are always smaller from C21 to C23 than from C23 to C21 for CS− trials. Similar conclusions are valid for (C1, C19) using GC method. Note that (C21, C23) and (C1, C19) have not been selected in Sessions S1 and S2, as they do not satisfy the inverse causality conditions using NC and GC, respectively.

**Table 2:** NC values of the pair (C21, C23) and GC values of the pair (C1, C19) in Gerbil 1.

	**CS+**		**CS−**	
**Session**	**$n_{C21\to C23}^{A}$ (10^−6^)**	**$n_{C23\to C21}^{A}$ (10^−6^)**	**$n_{C21\to C23}^{B}$ (10^−6^)**	**$n_{C23\to C21}^{B}$ (10^−6^)**
S3	1.1318	1.0218	0.5327	0.5631
S4	1.4024	1.2361	0.4163	0.5078
S5	1.1401	1.0943	0.3122	0.4232
S6	0.9102	0.8403	0.4060	0.4904
	$F_{C1\to C19}^{A}$	$F_{C19\to C1}^{A}$	$F_{C1\to C19}^{B}$	$F_{C19\to C1}^{B}$
S3	0.0178	0.0149	0.0118	0.0126
S4	0.0241	0.0184	0.0122	0.0145
S5	0.0264	0.0226	0.0145	0.0167
S6	0.0219	0.0147	0.0144	0.0147

For Gerbil 1, there are 6 *core pairs* for NC: (C21, C23), (C3, C18), (C17, C18), (C17, C22), (C17, C23), and (C21, C22), while we have 3 *core pairs* for GC analysis: (C1, C19), (C3, C22), and (C19, C21). The significance of some of the core pairs in the discrimination capability of neural signatures measured over the gerbil auditory cortex is studied next.

#### 3.1.3. Core Pairs for the Discrimination of CS− and CS+ Conditions

We define a classification rate for CS− and CS+ trials based on the inverse causality flow in a core pair. We use the following notations: *N*^*CS*+^ is the number of CS+ trials in a session; *N*^*CS*−^ is the number of CS− trials in a session; *N*^+^ is the number of CS+ trials with causality values satisfying Equations 1 or 2 for NC or GC, respectively; *N*^−^ is the number of CS− trials with causality values satisfying Equations 1 or 2 for NC or GC, respectively. For pair (C1, C2), the classification rates for CS+ and CS− conditions are given by $R_{\left(C1,C2\right)}^{+}=N^{+}/N^{CS+}$ and $R_{\left(C1,C2\right)}^{-}=N^{-}/N^{CS-}$, respectively. The overall classification rate is $R_{\left(C1,C2\right)}=\left(N^{+}+N^{-}\right)/\left(N^{CS+}+N^{CS-}\right)$. Using the above procedure, Table 3 displays the classification rates in Gerbil 1 for core pair (C21, C23) using NC and for core pair (C1, C19) using GC. For these core pairs, the mean classification rates are higher in Stage 2 than in Stage 1.

**Table 3:** Classification rates in Gerbil 1 using pairs (C1, C19) for GC and (C21, C23) for NC.

		**Stage 1**		**Stage 2**			
		**S1 (%)**	**S2 (%)**	**S3 (%)**	**S4 (%)**	**S5 (%)**	**S6 (%)**
NC	CS+	10.6	4.3	8.3	75.0	42.6	50
	CS−	89.1	89.1	95.8	89.6	91.5	80.9
	Overall	49.5	46.7	52.1	82.2	67.0	65.6
GC	CS+	85.1	78.2	70.8	91.6	68	78.2
	CS−	19.5	30.4	58.3	70.8	61.7	57.4
	Overall	52.6	54.3	64.5	81.2	64.8	67.7

Table 4 contains the average classification rates in Stages 1 and 2 for all 6 core pairs determined by NC method for Gerbil 1. We observe that the six core pairs show better classification performance in Stage 2 than in Stage 1 for NC method. The GC method gives the following three core pairs for Gerbil 1: (C1, C19), (C3, C22), and (C19, C21). Table 5 shows that two of the three GC core pairs have considerably better classification performance in Stage 2 than in Stage 1, while one pair has better classification in Stage 1. A comprehensive analysis involving all gerbils, however, shows that core pairs frequently produce worse classification in Stage 2 than in Stage 1. To characterize the corresponding effect, we will study a subset of core pairs with improved classification at Stage 2.

**Table 4:** Classification rates for the 6 core pairs obtained by NC method for Gerbil 1.

	**Stage 1**	**Stage 2**
	**Mean of S1 and S2 (%)**	**Mean of S3 **~** S6 (%)**
(C21,C23)	48.10	66.75
(C3,C18)	56.75	65.15
(C17,C18)	54.65	65.15
(C17,C22)	55.10	66.68
(C17,C23)	51.35	64.88
(C21,C22)	57.20	67.73

**Table 5:** Classification rates for the 3 core pairs obtained by GC method for Gerbil 1.

	**Stage 1**	**Stage 2**
	**Mean of S1 and S2 (%)**	**Mean of S3**~**S6 (%)**
(C1,C19)	53.55	69.63
(C3,C22)	55.70	66.20
(C19,C21)	44.30	32.75

### 3.2. Group Analysis of Emergent Cortical Activity Patterns

In this section, we introduce results of the causality analysis for Gerbils 2–7 in a similar way as previously for Gerbil 1 (see Supplementary Material for details). Here, we will study core pairs collected for all seven animals to obtain a comprehensive understanding of the emerging inverse causality flows following the switch to the avoidance phase. In particular, we study the common spatial patterns of the causality flows in the auditory cortex.

#### 3.2.1. Statistical Analysis of the Transition to Avoidance Phase

Results on the collected pairs with inverse causality flows for all seven gerbils are summarized in Table 6. Details of the tests of statistical significance using surrogate data are described in the Supplementary Material. We used the *d*′ criterion from signal detection theory with *d*′ > 1 to identify the transition from escape to avoidance strategy. According to this criterion, transitions occurred at S3 for Gerbil 1, then at S4, S2, S5, S2, S2, and S2 for Gerbils 2 though 7, respectively.

**Table 6:** Number of collected pairs using NC and GC in the experiments with 7 Gerbils^*^.

**Gerbil**	**Method**	**Session**						
**#**	**NC or GC**	**S1**	**S2**	**S3**	**S4**	**S5**	**S6**	**S7**
1	NC	11	18	**39**	**63**	**44**	**52**	
	GC	5	16	**25**	**56**	**43**	**30**	
2	NC	15	9	22	**27**	**29**		
	GC	16	5	29	**30**	**21**		
3	NC	11	**25**	**51**	**75**			
	GC	11	**43**	**61**	**78**			
4	NC	17	17	13	22	**25**	**28**	**25**
	GC	17	17	20	17	**30**	**33**	**17**
5	NC	8	**38**	**27**	**34**	**16**	**29**	
	GC	11	**50**	**22**	**40**	**23**	**24**	
6	NC	9	**12**	**28**	**16**	**23**		
	GC	6	**11**	**30**	**14**	**26**		
7	NC	3	**21**	**10**	**7**			
	GC	1	**14**	**4**	**9**			

*Bold numbers indicate session beyond the transition based on CD criteria*.

The number of collected pairs in any session of Stage 1 was smaller than the number of collected pairs in any session of Stage 2 in the case of NC. The collected pairs showed generally similar behavior using the GC analysis, with the exception of Gerbils 2 and 4 in some sessions toward the end of Stage 2. Still, the average number of collected pairs was larger in Stage 2 than in Stage 1 also for GC method.

In order to test the statistical significance of the difference in the number of collected pairs between Stages 1 and 2, we applied a paired group analysis using non-parametric Wilcoxon test, when all animals were pooled together for Stage 1 and Stage 2, respectively. The Wilcoxon test showed that the differences in the collected pairs between Stage 1 and Stage 2 were indeed statistically significant. Namely, for NC: *mean*_*Stage*1_ = 12.4 ± 1.2 (SEM), *mean*_*Stage*2_ = 30.1 ± 5.7 (SEM); Wilcoxon signed-rank test (W = 1), *p* < 0.05. For GC: *mean*_*Stage*1_ = 11.5 ± 1.6 (SEM), *mean*_*Stage*2_ = 30.0 ± 6.4 (SEM), Wilcoxon signed-rank test (W = 1), *p* < 0.05. The statistical t-test has limitations in our experiments due to the small number of data points, nevertheless, *t*-test also points to the significance of the difference between Stage 1 and Stage 2 in terms of collected pairs.

#### 3.2.2. Group Analysis of the Spatial Distribution of Collected and Core Pairs

We pooled channels that were part of collected pairs (in Stage 1) and core pairs (in Stage 2) for all animals where the array had a similar orientation with respect to the cortical surface, i.e., Gerbils 2, 4, 6, and 7 (see Supplementary Material). The spatial distribution of the corresponding electrodes are given in Table 7 for the pooled animals Gerbil 2, 4, 6, and 7. The numbers in Tables 7A,B indicate how many times a particular channel was counted as part of a collected pair in Stage 1 using (Table 7A) NC and (Table 7B) GC method, while Tables 7C,D shows the number of times a particular channel was counted as part of a core pair in Stage 2 using (Table 7C) NC and (Table 7D) GC method.

**Table 7:** The number of times a given channel is used as part of a collected pair in Stage 1 and as part of a core pair in Stage 2 for NC and GC.

**(A)**					**(B)**				
**Stage 1 (NC)**					**Stage 1 (GC)**				
1	4	0	3	6	0	11	3	7	20
24	14	14	20	15	20	15	12	14	19
16	14	16	16	16	14	21	16	10	14
28	10	18	13	16	24	11	19	16	16
**(C)**					**(D)**				
**Stage 2 (NC)**					**Stage 2 (GC)**				
1	2	0	0	5	3	1	1	2	6
6	1	3	2	2	5	5	9	6	2
3	6	8	3	5	3	11	5	5	5
4	4	5	1	5	5	4	3	3	6

**Figure 2:**
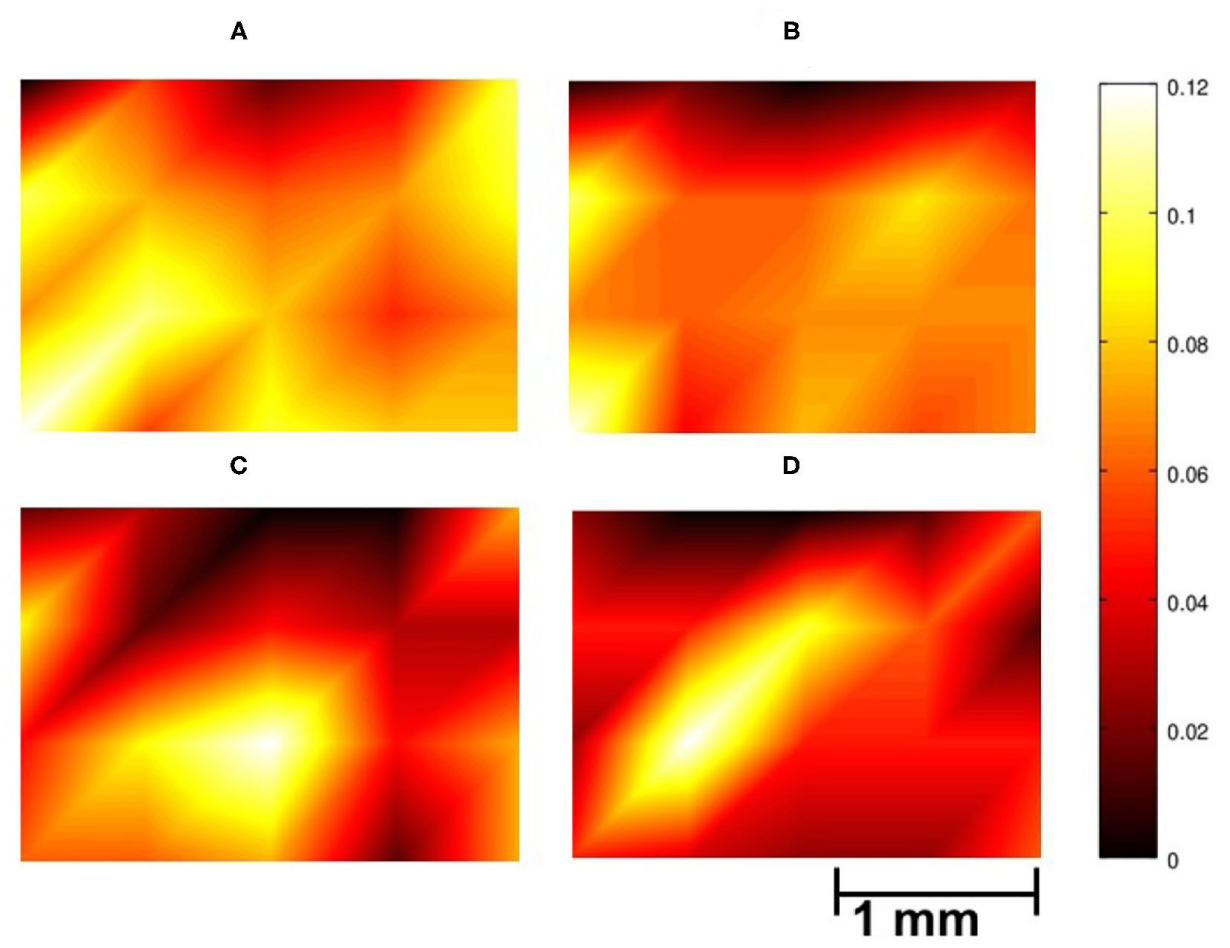
Spatial distribution of the contribution of the electrodes to inverse causality relationships over the auditory cortex of gerbils; illustrating data from a rectangular array of size 1.8 × 2.5 mm with 4 × 5 electrodes. The colorbar indicates the proportion of a specific channel appearing in the inverse causality pairs. **(A,B)** Case of collected pairs in Stage 1 using NC **(A)** and GC **(B)**; **(C,D)** Electrodes contributing to core pairs in Stage 2 for NC **(C)** and GC **(D)**. The bright regions around electrodes 23, 2, and 8 in **(C)**, and around 2 and 6 in **(D)** indicate the increased significance of those local areas in avoidance learning for these gerbils. To create the figures, piecewise linear interpolation is applied between the data points using MATLAB *interp* function.

To help visualize the spatial distribution of the pairs, Figure 2 provides a color coded representation of data in Table 7, where light yellow and deep purple colors mark high and low values, respectively. Data in Figure 2 have been obtained by normalizing each map with the total number of channel pair counts across the array. The distribution of collected pairs across the array in Stage 1 is rather uniform spatially for both NC and GC, without the emergence of a salient localized spatial clusters of channels with high degree of connectivity across the array (see Figures 2A,B). If the formation of pairs would be biased by the spatial neighborhood relationships, then channels in the middle of the array would be preferred as they have more neighbors that are physically close to them. This is not what we see in Figures 2A,B and in Table 7, where the nodes with the highest connectivity are in fact located at the boundary of the arrays; see first column of Table 7A and first and last columns of Table 7B.

For core pairs in Stage 2, on the other hand, there are localized spatial “hubs” for NC (electrodes 23, 2, and 8) and GC (electrodes 2 and 6; see Figures 2C,D). Moreover, the variance of the spatial distribution is higher in Figures 2C,D than in Figures 2A,B. Overall, we observe a marked difference between Stage 1 and Stage 2 based on the spatial distribution of causality relationships between channels. This result indicates that causal pairs can be useful tools to trace the neural basis underlying the transition from the escape strategy to the avoidance strategy.

## 4. Discussion and Interpretation of the Results

### 4.1. Significance of the Experimental Findings

We applied causality analysis to ECoG data recorded from seven Mongolian gerbils, which perform an auditory Go/NoGo discrimination task. We investigated how pairwise causal interactions between channels of the surface electrode array were affected by the progression of the learning. We studied inverse causality flows for CS+ and CS− stimuli, using the well-established Granger Causality (GC) and the recently introduced New Causality (NC). Our main findings are summarized as follows:

1. The number of channel pairs, which were collected based on the inverse causality criteria using GC and NC metrics significantly increased after the animals made a transition from the escape strategy (Stage 1) to the avoidance strategy (Stage 2) during the learning process.
2. Spatial patterns of *core pairs* have been analyzed during the learning process. It has been observed that the core pairs are more broadly distributed before transition to avoidance, while some regional hot spots emerged after the transition. This may imply that the implicated local areas play an important role in FM-tone discrimination during the learning process. This observation is especially prominent for NC analysis, while GC measure is more broadly distributed with less evidence of learning-related clustering.
3. The core pairs have been evaluated for classification performance regarding CS+ and CS− conditions. Some of the core pairs demonstrated statistically significant discrimination power after the transition to avoidance strategy, while most of the pairs did not possess such a property. These results require further detailed studies.

Our data with ECoG recordings in small rodents show that both NC and GC are capable to reliably track the development of causal interactions in primary sensory areas during learning. We found that NC and GC are comparable in terms of identifying the increase of the absolute numbers of collected channel pairs after the switch to the avoidance strategy. When applying the core pairs for classification of CS+ and CS− stimuli, NC appears to be a more robust method with respect to extracting common features expressed at the inter-individual level. Hence, our study supports earlier reports which point out advantages of NC over GC in the analysis of neuronal data based on human EEG (Hu et al., 2011, 2012, 2015), and extends these findings to ECoG recordings in small rodents.

In the present study we have focused on the gamma frequency band. We note that other frequency bands play also an important role in grouping and segregating neuron assemblies and consequently encode stimulus identification, memory and drive (goal directed) behavior. However, signals in the theta band are prone to contamination of volume-conducted electrical activity generated by distal sources e.g., hippocampus, particularly during locomotion (Lalla et al., 2017). Delta bands might be affected by entrained neuronal activity caused by the inter-stimulus interval of 2 Hz of the FM-tones in the tone sequences in the present study. On the other hand, local gamma band synchrony seems to drive discrimination learning. Concina et al. (2018) found an increased coherence of slow gamma band activity between prelimbic and auditory cortex only for animals which did learn an auditory discrimination task. This increased coherence was absent for other frequency bands (theta, beta) and non-learning animals.

Our results contribute to the ongoing discussion on the role of neuronal assembly interactions for auditory learning and their qualitative nature (Eggermont, 2006; Grosso et al., 2015; Keating and King, 2015; Ohl, 2015; Weinberger, 2015). For instance, the behavioral relevance of temporal coherence or coupling for auditory information processing has been demonstrated by numerous recent studies both in intra-cortical circuits (Noda et al., 2014; Yokota et al., 2015), as well as on cortico-subcortical circuit levels (Bachiller et al., 2014; Liégeois-Chauvel et al., 2014; Schulz et al., 2016). In this context, our finding of an increase in inverse predictive causality flow among certain channel pairs with the progression of learning can be reasonably interpreted as an indication of spatial reorganization or remapping of effective intra-cortical connectivity on the mesoscopic level of neuronal assemblies (Weinberger and Bakin, 1998; Pienkowski and Eggermont, 2011).

The criterion of inverse causality flow seems particularly suited in revealing the potential reorganization of intra-cortical interactions during learning, because it emphasizes qualitative changes in the directional component of pairwise interactions that emerge over the course of the training, as opposed to quantitative changes in the absolute magnitude of unidirectional causal flows. These findings emphasize the importance of functional connectivity changes in unimodal sensory areas, including the primary auditory cortex, as potential neural contributors to behavioral changes.

### 4.2. Evolving Graph Model of Dynamic Cortical Activity Patterns

We introduce a graph theoretical model featuring phase transition behavior, to interpret learning effects in the gerbil experiments. We develop a random graph to model the cortical neuropil with short- and long-range connections (edges) describing the combination of short dendritic connections and long axons (see Kozma et al., 2005, 2012; Kozma and Freeman, 2016). The short dendritic connections are modeled by lattice edges on a torus, while long axons are described by a few additional edges superimposed on the lattice. This model is related to small-world networks, and it provides a systematic treatment of some of their properties related to cortical networks (see Bollobás et al., 2010; Bullmore and Sporns, 2012; Haimovici et al., 2013). The definition of the model captures an important property of cortical networks; namely, it is more likely for a neuron to be connected to nearby neurons than to neurons which are far from the given one. The long-range connections have power law length distributions, leading to drastic reduction of the diameter of the brain graph (see Janson et al., 2019).

#### 4.2.1. Bootstrap Percolation on $G_{{\mathbb{Z}}_{N}^{2},p_{d}}$

The spread of activity in the cortical tissue will be described by a simplified mathematical model—bootstrap percolation (BP) (see, e.g., Balister et al., 2006; Bollobás et al., 2010). The model is defined as follows. The set of vertices of the random graph $G_{{\mathbb{Z}}_{N}^{2},p_{d}}$ is given by the vertex set of ${\mathbb{Z}}_{N}^{2}$, where ${\mathbb{Z}}_{N}^{2}$ is a torus on *N*^2^ vertices. All edges of the torus ${\mathbb{Z}}_{N}^{2}$ are presented in $G_{{\mathbb{Z}}_{N}^{2},p_{d}}$. The random edges of $G_{{\mathbb{Z}}_{N}^{2},p_{d}}$ are distance-dependent, i.e., the probability that an arbitrary pair of vertices, *u, v*, that are at graph distance *d* apart of each other, is given by

(3) $$\begin{array}{r} p_{d}=\mathbb{P}\left(\left(u,v\right)\in E\left(G_{{\mathbb{Z}}_{N}^{2},p_{d}}\right)|dist\left(u,v\right)=d\right)=\frac{c}{Nd^{\alpha }}, \end{array}$$

where *d* > 1, *c* is a positive constant, and α is the power exponent of long-range edge length distribution. In this work, we use α = 1, for simplicity. It is assumed that there are no multiple edges between the vertices. We will use the following notation λ = 4 c ln 2, i.e., λ is proportional to the probability of adding long-range edges.

We state here a generalized definition of bootstrap percolation that consists of two types of vertices, which correspond to excitatory and inhibitory units. Bootstrap percolation with one type of vertices (excitatory or inhibitory) can be viewed then as a special case. At the beginning each vertex of the graph is described by two random variables, its type and state. The types of vertices are excitatory (1) or inhibitory (2). We define the type as a Bernoulli random variable *Be*(ω), which is selected at the start of the process and remains unchanged afterwards. In contrast, state of a vertex may change during the process as follow. At each time step a vertex is either active or inactive. For each vertex *v* of the graph we assign a binary function χ_*v*_(*t*) which describes the activity state of the vertex at time *t*. A vertex is said to be active if χ_*v*_(*t*) = 1, otherwise it is inactive and χ_*v*_(*t*) = 0. For each vertex *v*, the potential function χ_*v*_(0) at the beginning is a Bernoulli random variable Be(*p*).

Let $A_{i}\left(t\right)=\left\{v\in V\left(G_{{\mathbb{Z}}_{N}^{2},p_{d}}\right)|\chi_{v}\left(t\right)=1\&v\text{ is of type}\text{i}\right\}$, *i* ∈ {*E, I*}, denote the set of active vertices of type *E* and *I* at time *t*, respectively, *A*(*t*) = *A*_*E*_(*t*)∪*A*_*I*_(*t*). *A*(0) consists of all vertices that are active at the beginning. Each vertex may change its activity based on the states of its neighbors. For a vertex *v* which is of type *E*, the evolution rule is given by

(4) $$\begin{array}{r} \chi_{v}\left(t+1\right)=𝟙\left(\underset{u\in N^{E}\left(v\right)}{\sum }\chi_{u}\left(t\right)-\underset{u\in N^{I}\left(v\right)}{\sum }\chi_{u}\left(t\right)\geq k_{1}\right), \end{array}$$

where *N*^*E*^(*v*) and *N*^*I*^(*v*) denote the subset of vertices in the closed neighborhood of vertex *v*, which are of *E* and *I* type, respectively; and 𝟙 is the indicator function. For a vertex *v* of type *I*, the following rule holds

(5) $$\begin{array}{l} \chi_{v}(t+𝟙)=𝟙\left(\sum_{u\in N^{E}(v)}\chi_{u}(t)+\sum_{u\in N^{I}(v)}\chi_{u}(t)\geq k_{2}\right)\\ \;=𝟙\left(\sum_{u\in N(v)}\chi_{u}(t)\geq k_{2}\right), \end{array}$$

where *N*(*v*) = *N*^*E*^(*v*)∪*N*^*I*^(*v*) is the closed neighborhood of vertex *v*. The rules try to capture the behavior that a node is active if its neighbors total activity (considering sign) exceeds a threshold *k*_1_ or *k*_2_, respectively.

We consider the evolution of the following two density functions, corresponding to active nodes of type 1 (excitatory) and type 2 (inhibitory). Let:

(6) $$\begin{array}{r} \rho_{t}^{\left(1\right)}=\frac{\left|A_{1}\left(t\right)\right|}{\omega N^{2}} \end{array}$$

and

(7) $$\begin{array}{r} \rho_{t}^{\left(2\right)}=\frac{\left|A_{2}\left(t\right)\right|}{\left(1-\omega \right)N^{2}} \end{array}$$

be the densities of the first and second types, correspondingly, where *A*_*i*_(*t*) is the number of active vertices of type *i*, *i* = 1, 2, at time *t*. Then, in particular, the density of all active nodes is given by

$$\rho_{t}=\omega \rho_{t}^{\left(1\right)}+\left(1-\omega \right)\rho_{t}^{\left(2\right)}=\frac{\left|A_{1}\left(t\right)|+|A_{1}\left(t\right)\right|}{N^{2}}.$$

The dynamics of the BP with one type of (excitatory) vertices is fully described in Janson et al. (2019).

**Theorem 1**. *In the mean-field approximation of the activation process *A*(*t*) over random graph $G_{{\mathbb{Z}}_{N}^{2},p_{d}}$ there exists a critical probability *p*_*c*_ such that for a fixed *p*, with high probability for large *N*, all vertices will eventually be active if *p* > *p*_*c*_, while all vertices will eventually be inactive for *p* < *p*_*c*_. The value of *p*_*c*_ is given as the function of *k* and* λ *as follows:*

- *For* *k* = 0 *and any* λ, *p*_*c*_ = 0 *and all vertices will become active in one step for any* *p*.
- *For* *k* = 1 *and any* λ, *p*_*c*_ = 0, *i.e., for any fixed* *p* > 0, *all vertices will eventually become active with high probability*.
- *For* *k* = 2, 3, 4 *and any* λ, *p*_*c*_ = *x*_*k*_(λ), *where* *x*_*k*_(λ) ∈ (0, 1) *is a nontrivial solution to* $x={\overset{-}{f}}_{k}\left(x\right)$.
- *For* *k* = 5 *and* λ > ln(5), *p*_*c*_ = *x*_5_(λ), *where* *x*_5_(λ) ∈ (0, 1) *is a nontrivial solution to* $x={\overset{-}{f}}_{5}\left(x\right)$; *for* λ ≤ ln(5), *p*_*c*_ = 1.

It is possible to describe the dynamics of BP with two types of (excitatory and inhibitory) nodes (Kozma et al., 2016b). In this case, one needs to find the solutions of the following set of fixed-point equations, for the mathematically derived nonlinear mapping functions *f*_1_(*x, y*) and *f*_2_(*x, y*):

(8) $$\left\{ \begin{array}{l} f_{1}(x,y)=x,\\ f_{2}(x,y)=y. \end{array}\right.$$

**Figure 3:**
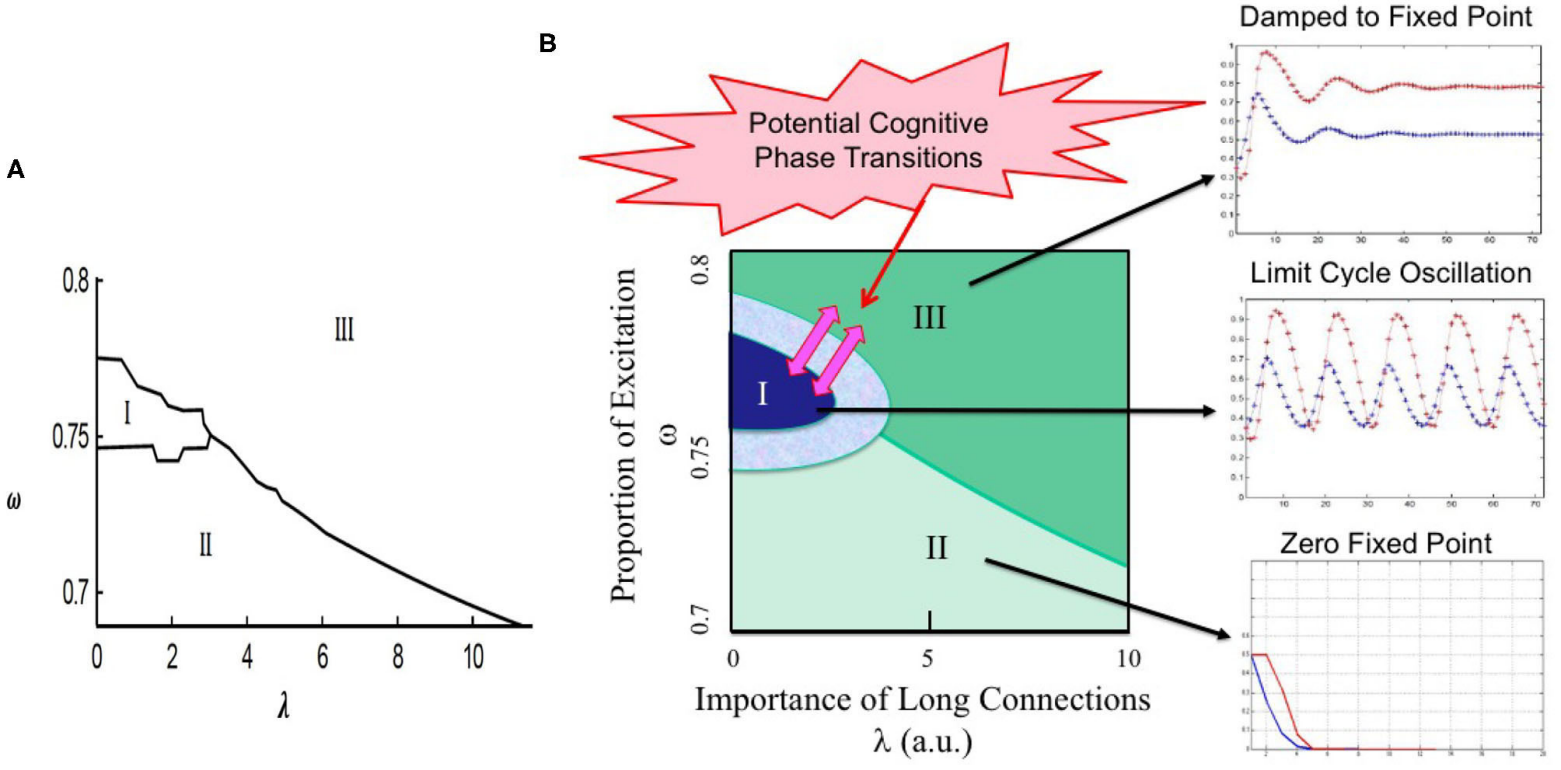
Illustration of the potential of bootstrap percolation (BP) model to describe learning-induced state transitions in the sensory cortex. **(A)** Bifurcation diagram of BP with two types of nodes (excitatory and inhibitory), where *k*_1_ = 2 and *k*_2_ = 3. Parameter ω gives the proportion of excitatory neurons; increasing λ signifies stronger long-range connections. Limit cycle dynamics exists in region I, processes of both types eventually die out in II, and non-zero fixed point dynamics exist in region III; **(B)** Proposed interpretation of dynamic regimes illustrated over the phase diagram in the λ vs. ω space. The solid dark (blue) region corresponds to conditions with limit cycle oscillations created as the result of learning. There is a transitionary region (light blue) illustrate transitionary conditions due to local parameters and inhomogeneities. Transitions from limit cycle regime to non-zero base state (dark green area) indicate the absence of learnt stimuli. Zero fixed point (light green) is shown in the lower segment of the phase diagram. The region corresponding to the hypothetic cognitive phase transitions is illustrated by pink arrows; modified and reprinted with permission from Kozma et al. (2016b).

The fixed point can be either stable or unstable, which determines the nature of the percolation dynamics, e.g., fixed point, limit cycle, or non-periodic oscillations (Kozma et al., 2016b). Numerical examination of BP with two types of vertices shows dynamic behavior that is richer than in BP with only one type of vertices. In particular, when the threshold parameter in the update rules for vertices of the first and second types are different, limit cycle dynamics may appear. A bifurcation diagram of the process with excitatory-inhibitory nodes is shown in Figure 3A with respect to ω, λ, where parameters *k*_1_ = 2 and *k*_2_ = 3. The type of a vertex is determined at the beginning uniformly at random, thus ω defines the overall ratio of excitatory and inhibitory nodes, which is unchanged throughout the process. It is observed that limit cycle behavior belongs to the excitatory to inhibitory ratio of around 4/1. The next section will investigate how the phase transitions to narrow-band (limit-cycle) oscillations in the percolation model may be beneficial in the interpretation of the experimental findings on the emergence of common-mode oscillations in neural populations as the result of the learning process.

### 4.3. Graph Models for Describing Learning-Induced Phase Transitions

In this section we analyze the potential role of several key model parameters in the interpretation of the learning dynamics of the cortical tissue. Based on Freeman's neurodynamics theory, we know that learning is closely related to the onset of narrow band oscillations in the cortex as a consequence of sustained reinforcement signal, and the possible formation of Hebbian cell assemblies (Freeman, 1991). These insights provide important input to our modeling studies, which provide theoretical insights into the interpretation of the experimental results.

Our bootstrap percolation model describes phase transitions between fixed point and limit cycle dynamics when certain control parameters of the model vary. Here let us focus on the significance of long-range edges (λ) and threshold values of the update rule (*k*_1_ and *k*_2_). For example, in the case of *k*_1_ = *k*_2_ = 3 our model shows fixed point behavior, which can change to oscillatory behavior if *k*_1_ is decreased to 2. Decreasing *k*_1_ can be interpreted as the reduction of the dynamic threshold in the interaction between the cortical locations due to learning. Moreover, changes in λ can also produce transitions between dynamic regimes. This is illustrated in Figure 3B, which is based on the phase diagram obtained from our model and depicted in Figure 3A. Three main regions are shown as follows: Region I: limit cycle oscillations at the middle range of ω (deep blue); Region II: zero fixed point regime (light green) at the bottom section; Region III: nonzero fixed point (dark green) at the top half. The region corresponding to the hypothetic cognitive phase transitions is indicated by pink arrows (pink).

The presence of the oscillatory behavior in a narrow frequency band is shown in Figure 3B using Region I (dark blue). Oscillatory behavior is a hallmark of learning effects, which are modeled in our model using the adaptation (decrease) of excitatory threshold *k*_1_ from its original value of 3 to 2, while the inhibitory threshold *k*_2_ remained unchanged; the corresponding effect is visualized by the transitionary region with light blue color around Region I. Note, that in the case of high values of both excitatory and inhibitory thresholds *k*_1_ = *k*_2_ = 3 no oscillatory behavior takes place, rather the dynamics converges to fixed points (not shown). In short, *k*_1_ may be indeed a control parameter for a state transition in neurodynamics accompanying a cognitive state transition during learning.

The results introduced in this study show that it is possible to detect neural correlates of cognitive state changes in the gerbil's cortex using causality measures. The difference between the behavior of the early escape stage and advanced avoidance strategy in the trained animal has an experimentally observable neural measure through the formation of connections (causal links) between certain cortical regions as the result of learning. The regions and links between them may be the manifestations of Hebbian cell assembly (HCA) formation, and our results may provide evidence for the existence of HCAs.

## 5. Dedication

This work is dedicated to the memory of our dear colleague and friend Sanqing Hu, who relentlessly pursued research at the forefront of science, leading to the discoveries reported in this work. Without him these results would not exist.

## Data Availability Statement

The data supporting the conclusions of this article will be made available on reasonable requests to the corresponding authors, without undue reservations.

## Ethics Statement

The animal study was reviewed and approved by Ethics Commission of the state of Sachsen-Anhalt, Germany. All experiments were in compliance with the guidelines of the European Community (EUVD 86/609/EEC).

## Author Contributions

The study was conceived by FO and RK. Experiments and data preprocessing were conducted by AG, FO, AS, TW, and MW. SH contributed to the overview of causality analysis. YS contributed to data analysis and computational algorithms design. MR, YS, and RK contributed to graph theory modeling. YS and SH leading the HDU team conducted the computer analysis for causality. All authors contributed to interpretation of the data. RK and FO coordinated the interdisciplinary research team. RK, FO, and AS wrote the manuscript that was approved by all authors.

## Conflict of Interest

The authors declare that the research was conducted in the absence of any commercial or financial relationships that could be construed as a potential conflict of interest.
