# Supplementary material for: State Transitions During Discrimination Learning in the Gerbil Auditory Cortex Analyzed by Network Causality Metrics
Source: Front Syst Neurosci. 2021 Apr 22;15:641684. doi: 10.3389/fnsys.2021.641684 (PMC8100519; doi:10.3389/fnsys.2021.641684)
Supplement: Supplementary file 1 [file Image_1.pdf]

Supplementary Material

All experiments were in compliance with the guidelines of the European Community (EUVD 86/609/EEC) and were approved by an ethics commission of the state of Sachsen-Anhalt. Seven adult male Mongolian gerbils (*Meriones unguiculatus*) (75 - 105 g) were used for the experiments described here.

0.1 Surgery and Electrodes

Implantation procedures were adapted from our previous work. Ohl et al. (2000b); Woldeit et al. (2012). Briefly, animals were induced by intraperitoneal infusion of a mixture of ketamine (100 mg per kg body weight, Ratiopharm, Germany) and xylazine (40 mg per kg BW, Rompun, Bayer, Germany). Further anesthetic was given as necessary to maintain areflexia. After onset of anesthesia, the right temporal muscle was removed and a craniotomy of approximately 4 mm diameter was performed over the right auditory cortex (center: bregma -3 mm; lateral 5.8 mm). A custom-made rectangular 5×4 electrode array (stainless steel wires, diameter $76.2\ \mu m$, impedance: $50 - 500\ k\Omega$, interelectrode distance: $600\ \mu m$) was centered over the epidural surface of primary auditory cortex using previously established stereotactic landmarks; Thomas et al. (1993); Ohl et al. (2000b,a). The array was fixed with biocompatible UV-glue (Plurafill flow, Pluradent, Germany). A stainless steel screw (winding diameter 1 mm, length 2 mm, FST) connected to a silver wire diameter $127\ \mu m$) was implanted over the left visual cortex (approximately bregma -5 mm and lateral 2.5 mm) and served as reference. The numbering of the 20 electrodes on the 4×5 array is illustrated in Fig.S2. Animals were allowed to recover from surgery for one week prior to experiments.

28	12	26	10	24
8	22	6	20	4
18	2	23	7	21
5	19	3	17	1

Figure S1: Spatial arrangement of the surface electrode array showing the location of the individual channels. Channel numbers are based on the digital output of the 32 channel recording system; the nonconsecutive order of channel indices is due to the customized electrode plugs.

0.2 Discrimination Learning Paradigm

Gerbils were trained in a two-way active avoidance paradigm (Go/NoGo) in a shuttle box Wetzel et al. (2008); Schulz et al. (2016) (shuttle box system $35 \times 20 \times 30\ cm$; Coulbourne Instruments, USA) located inside a soundproof Faraday cage (Industrial Acoustics Company, Germany). Animals were exposed to two different types of tones: a sequence of rising frequency modulated tones (FM 2-4 kHz, duration 200 ms, inter-tone onset interval 500 ms) which constituted the CS^+ (Go response expected) and a sequence of falling FM tones (4-2 kHz) which constituted the CS^- (NoGo response expected). Tones were delivered by a loudspeaker positioned above the shuttle box calibrated to a sound pressure level of approximately 65

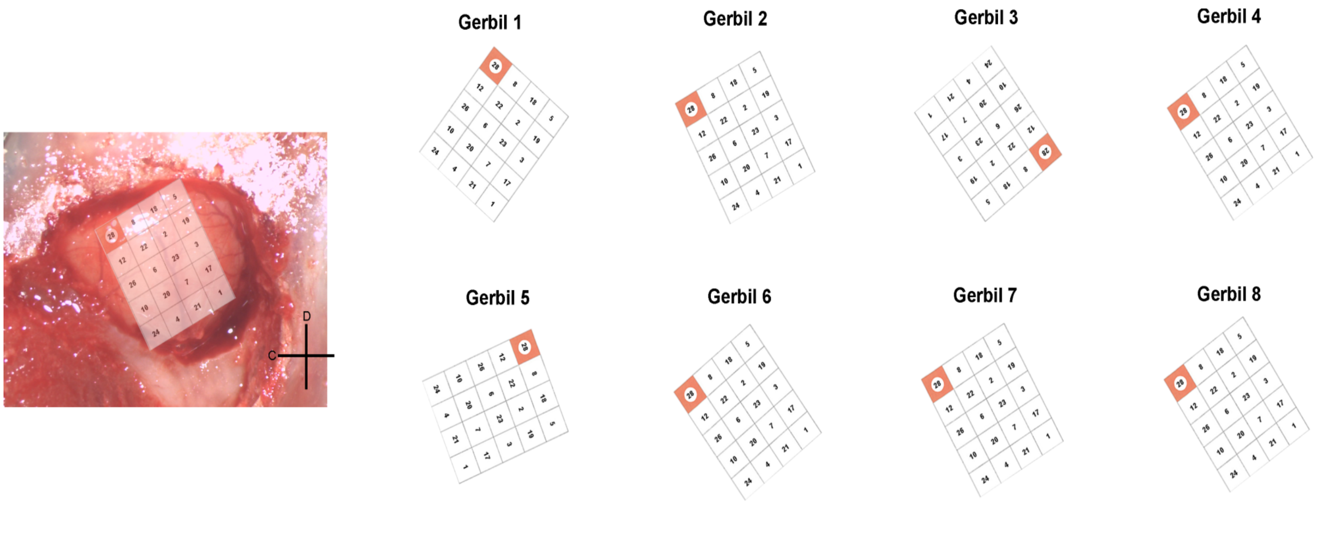

(a) (b)

Figure S2: Arrangement of the arrays over the auditory cortex. (a): Exposed surface of the right auditory cortex after craniotomy in one example gerbil with the semitransparent scheme of the electrode array superimposed. (b): Array orientation with respect to the cortical surface as shown in (a) for gerbils 1 to 8. Note that for Gerbils 2, 4, 6, 7 and 8, the orientation of the array was very similar, whereas the orientation of the array was rotated by 90 degrees for Gerbils 1 and 5 and by 180 degrees for Gerbil 3.

dB as measured inside the shuttle box. Animals had to learn to shuttle to the contralateral compartment in response to the CS+ within a period of 6 s in order to avoid a mild electrical foot shock applied via the metal grid floor (300 μA). In contrast, animals had to learn to stay within the ipsilateral compartment in response to the CS- for a period of at least 10 s. Successful shuttling in response to the CS+ was scored as a *hit* whereas failure of shuttling in response to the CS- was scored as a *miss*. In turn, incorrect shuttling in response to the CS- was scored as a *false alarm*, whereas successful avoidance of shuttling in response to the CS- was scored as a *correct rejection*, respectively.

In five out of seven animals (Gerbils 1-5), both miss and false alarm responses were reinforced by the foot shock (symmetric reinforcement), whereas in two animals (Gerbils 6-7) only *miss* trials were reinforced (asymmetric reinforcement). Training was conducted in daily sessions of 48 CS+ and 48 CS- trials presented in pseudo-randomized order (Gellermann sequence) with an inter-trial interval of 25 - 30 s. We determined the quantity d', which quantifies detection sensitivity independent of the response bias of the animal. Deliano et al. (2009); Happel et al. (2014) A value $d' \geq 1$ usually corresponds to significant discrimination above chance level. Training sessions were continued until the animal reached the performance criterion defined by value $d' \geq 1$ in at least three sessions, including two consecutive sessions. After removing bad channels and bad trials, the number of trials and channels analyzed for the gerbils are summarized in Table S1.

0.3 Electrophysiology and Pre-processing of Data

Electrocorticograms (ECoGs) were recorded during the training sessions via a 32 channel recording system (Tucker-Davis Technologies, USA), hardware filtered between 0.1 - 300 Hz and digitized at 1 kHz. For causality analysis, the ECoG was band-pass filtered in the gamma range (20-80 Hz). ECoG segments of duration 2.5 s were selected for all CS+ and CS- trials, starting from the trial onset, i.e. from the start

of the CS+/CS- FM-tone sequence. The segment length of 2.5 s was chosen to maximize the number of segments without movement (e.g. before shuttling during a hit trial) or shock artifacts (after incorrect shuttling during a false alarm trial). Segments and channels that contained visible artifacts of any kind (bad channels) were excluded from the analysis.

0.4 Causality Analysis: Granger Causality (GC) and New Causality (NC)

Here the methodology established by Hu et al. (2011) is summarized, starting with the basics of Granger Causality and extending to New Causality. Consider two stochastic time series which are assumed to have stationary joint distribution. We develop autoregressive representation models for the processes as follows Box et al. (2011):

$$\begin{cases} X_{1,t} = \sum_{j=1}^{m} \mathbf{a}_{11,j} X_{1,t-j} + \epsilon_{1,t}, \\ X_{2,t} = \sum_{j=1}^{m} \mathbf{a}_{22,j} X_{2,t-j} + \epsilon_{2,t}. \end{cases} \tag{S1}$$

Their joint representations are described as

$$\begin{cases} X_{1,t} = \sum_{j=1}^{m} a_{11,j} X_{1,t-j} + \sum_{j=1}^{m} a_{12,j} X_{2,t-j} + \eta_{1,t}, \\ X_{2,t} = \sum_{j=1}^{m} a_{21,j} X_{1,t-j} + \sum_{j=1}^{m} a_{22,j} X_{2,t-j} + \eta_{2,t}, \end{cases} \tag{S2}$$

where $t = 0, 1, \cdots, N$, the noise terms ϵ_i and η_i are uncorrelated over time and have zero means and variances of $\sigma^2_{\epsilon_i}$, and $\sigma^2_{\eta_i}$, $i = 1, 2$. The covariance between η_1 and η_2 is denoted by $\sigma_{\eta_1\eta_2} = cov(\eta_1, \eta_2)$. Hu et al. (2011) A general approach to determine the optimal order of the Multivariate Autoregressive model is given by the Akaike Information Criterion (AIC) Akaike (1974). In this work, we tested various values of model order m with the actual data. Finally, we set $m = 10$ in all experiments, and values $m > 10$ lead to similar results.

0.5 GC in Time Domain

We consider the first of the two equations in Eqs. (S1) and (S2), respectively. If $\sigma^2_{\eta_1}$ is less than $\sigma^2_{\epsilon_1}$, X_2 is said to have a causal influence on X_1. In this case, the first equation in Eq. (S1) more accurately approximates X_1 than the first equation in Eq. (S2). Otherwise, if $\sigma^2_{\eta_1} = \sigma^2_{\epsilon_1}$, X_2 is said to have no causal influence on X_1. The corresponding causal influence is called Granger Causality (GC), Geweke (1982); Ding et al. (2006), defined as follows:

$$F_{X_2 \to X_1} = \ln \frac{\sigma^2_{\epsilon_1}}{\sigma^2_{\eta_1}}. \tag{S3}$$

Moreover, there is causal influence from X_2 to X_1, if $F_{X_2 \to X_1} > 0$, while there is no causal relationship if $F_{X_2 \to X_1} = 0$. Similarly, the causal influence from X_1 to X_2 is defined by:

$$F_{X_1 \to X_2} = \ln \frac{\sigma^2_{\epsilon_2}}{\sigma^2_{\eta_2}}. \tag{S4}$$

0.6 NC in Time Domain

Based on the first equality in Eq. (S2), we can see that contributions to $X_{1,t}$ include terms $\sum_{j=1}^{m} a_{11,j}X_{1,t-j}, \sum_{j=1}^{m} a_{12,j}X_{2,t-j}$ and the noise term $\eta_{k,t}$, where the influence from quantity $\sum_{j=1}^{m} a_{11,j}X_{1,t-j}$ is causality from X_1's own past values. Each contribution plays an important role in determining $X_{1,t}$. If $\sum_{j=1}^{m} a_{12,j}X_{2,t-j}$ occupies a larger portion among all those contributions, then X_2 has stronger causality on X_1, and vice versa. A refined definition for causality from X_2 to X_1 in time domain should be able to describe what proportion X_2 occupies among all these contributions. Based on these considerations, NC from X_2 to X_1 is defined by Hu et al. (2011) as follows:

$$n_{X_2 \to X_1} = \frac{\sum\limits_{t=m}^{N} \left(\sum\limits_{j=1}^{m} a_{12,j}X_{2,t-j}\right)^2}{\sum\limits_{h=1}^{2} \sum\limits_{t=m}^{N} \left(\sum\limits_{j=1}^{m} a_{1h,j}X_{h,t-j}\right)^2 + \sum\limits_{t=m}^{N} \eta_{1,t}^2}. \tag{S5}$$

Similarly, NC in time domain from X_1 to X_2 is defined by

$$n_{X_1 \to X_2} = \frac{\sum\limits_{t=m}^{N} \left(\sum\limits_{j=1}^{m} a_{21,j}X_{1,t-j}\right)^2}{\sum\limits_{h=1}^{2} \sum\limits_{t=m}^{N} \left(\sum\limits_{j=1}^{m} a_{2h,j}X_{h,t-j}\right)^2 + \sum\limits_{t=m}^{N} \eta_{2,t}^2}. \tag{S6}$$

0.7 Surrogate data for statistical significance analysis

To test the statistical significance of the pairwise interaction given by the $n_{X_1 \to X_2}$ and $F_{X_1 \to X_2}$ causality values regarding CS+ and CS- conditions, we employed a shuffling procedure Hu et al. (2012, 2015). Surrogate data were generated by randomly shuffling CS+ trials for channel X_1 of collected pair (X_1, X_2), then comparing the metrics produced by the original (unshuffled) and the surrogate (shuffled) data. This shuffling procedure has been repeated independently 100 times. The results are displayed in Fig.S3 showing that the causality indices determined using the original data have always exceeded indices based on the surrogate data in the case of NC, and most of the time for the case of GC as well. These observations demonstrate that the original data points for collected pair (X_1, X_2) were not produced by chance.

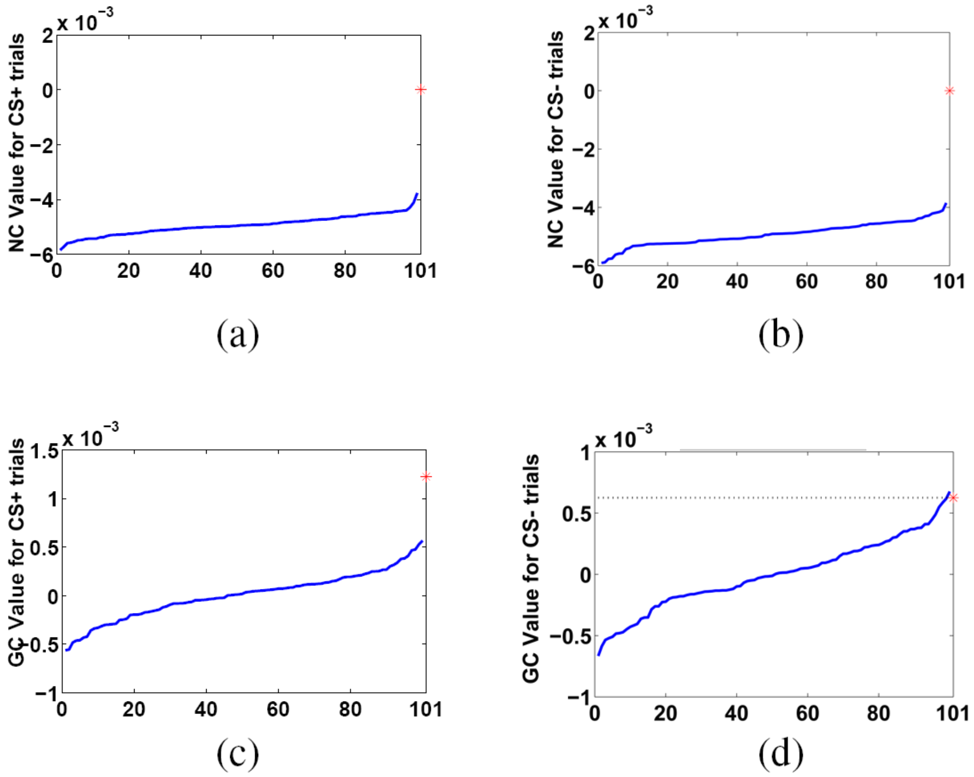

Figure S3: Significance test of the collected pair statistics using surrogate data in Gerbil 4. The red dots show the causality indices for the original data, while the blue lines show the values obtained using the surrogate data. (a): $n_{X_1 \to X_2}$ values for NC for CS+ case (Go is correct) with shuffling X_1; (b): $n_{X_1 \to X_2}$ values for NC for CS- case (NoGo is correct) with shuffling X_2; (c): $F_{X_1 \to X_2}$ values for GC for CS+ case (Go is correct) with shuffling X_1; (d): $F_{X_1 \to X_2}$ values for GC for CS- case (NoGo is correct) with shuffling X_2.

REFERENCES

Akaike, H. (1974). A new look at the statistical model identification. *IEEE transactions on automatic control* 19, 716–723

Box, G. E., Jenkins, G. M., and Reinsel, G. C. (2011). *Time series analysis: forecasting and control*, vol. 734 (John Wiley & Sons)

Deliano, M., Scheich, H., and Ohl, F. W. (2009). Auditory cortical activity after intracortical microstimulation and its role for sensory processing and learning. *Journal of Neuroscience* 29, 15898–15909

Ding, M., Chen, Y., and Bressler, S. (2006). Granger causality: Basic theory and application to neuroscience. *Handbook of Time Series Analysis [Internet]. Wiley, Weinheim*

Geweke, J. (1982). Measurement of linear dependence and feedback between multiple time series. *Journal of the American statistical association* 77, 304–313

Happel, M. F., Niekisch, H., Rivera, L. L. C., Ohl, F. W., Deliano, M., and Frischknecht, R. (2014). Enhanced cognitive flexibility in reversal learning induced by removal of the extracellular matrix in auditory cortex. *Proceedings of the National Academy of Sciences* 111, 2800–2805

Hu, S., Cao, Y., Zhang, J., Kong, W., Yang, K., Zhang, Y., et al. (2012). More discussions for granger causality and new causality measures. *Cognitive neurodynamics* 6, 33–42

Table S1. Overview of trials and channels analyzed in the experiments with 7 Gerbils

Gerbil #	Trial Type	Session							Number of Channels
		S1	S2	S3	S4	S5	S6	S7	
1	CS+	47	46	48	47	46	46		18
	CS-	46	43	47	47	47	47		
2	CS+	43	48	48	42	48			16
	CS-	38	47	48	45	47			
3	CS+	45	44	35	43				18
	CS-	46	36	43	40				
4	CS+	27	40	41	46	39	41	41	16
	CS-	27	28	32	43	26	29	28	
5	CS+	41	41	47	36	40	29		17
	CS-	42	43	43	40	34	31		
6	CS+	47	37	43	48	48			14
	CS-	47	47	46	48	45			
7	CS+	46	48	48	48				13
	CS-	48	45	46	48				

Hu, S., Dai, G., Worrell, G. A., Dai, Q., and Liang, H. (2011). Causality analysis of neural connectivity: critical examination of existing methods and advances of new methods. *IEEE transactions on neural networks* 22, 829–844

Hu, S., Wang, H., Zhang, J., Kong, W., Cao, Y., and Kozma, R. (2015). Comparison analysis: Granger causality and new causality and their applications to motor imagery. *IEEE transactions on neural networks and learning systems* 27, 1429–1444

Ohl, F. W., Scheich, H., and Freeman, W. J. (2000a). Topographic analysis of epidural pure-tone–evoked potentials in gerbil auditory cortex. *Journal of Neurophysiology* 83, 3123–3132

Ohl, F. W., Schulze, H., Scheich, H., and Freeman, W. J. (2000b). Spatial representation of frequency-modulated tones in gerbil auditory cortex revealed by epidural electrocorticography. *Journal of Physiology-Paris* 94, 549–554

Schulz, A. L., Woldeit, M. L., Gonçalves, A. I., Saldeitis, K., and Ohl, F. W. (2016). Selective increase of auditory cortico-striatal coherence during auditory-cued go/nogo discrimination learning. *Frontiers in behavioral neuroscience* 9, 368

Thomas, H., Tillein, J., Heil, P., and Scheich, H. (1993). Functional organization of auditory cortex in the mongolian gerbil (meriones unguiculatus). i. electrophysiological mapping of frequency representation and distinction of fields. *European Journal of Neuroscience* 5, 882–897

Wetzel, W., Ohl, F. W., and Scheich, H. (2008). Global versus local processing of frequency-modulated tones in gerbils: an animal model of lateralized auditory cortex functions. *Proceedings of the National Academy of Sciences* 105, 6753–6758

Woldeit, M., Schulz, A., and Ohl, F. (2012). Phase de-synchronization effects auditory gating in the ventral striatum but not auditory cortex. *Neuroscience* 216, 70–81
